# The adaptive value of camouflage and colour change in a polymorphic prawn

**DOI:** 10.1038/s41598-018-34470-z

**Published:** 2018-10-30

**Authors:** Rafael Campos Duarte, Martin Stevens, Augusto Alberto Valero Flores

**Affiliations:** 1 Centro de Biologia Marinha, Universidade de São Paulo, Rod. Manoel Hypólito do Rego, km 131.5, São Sebastião, SP 11612-109 Brazil; 2 Programa de Pós-Graduação em Biologia Comparada, Faculdade de Filosofia, Ciências e Letras de Ribeirão Preto, Universidade de São Paulo, Ribeirão Preto, Brazil; 3 Centre for Ecology and Conservation, University of Exeter, Penryn Campus, Penryn, TR10 9FE UK

## Abstract

Camouflage has been a textbook example of natural selection and adaptation since the time of the earliest evolutionists. However, aside from correlational evidence and studies using artificial dummy prey, experiments directly showing that better camouflaged prey to predator vision are at reduced risk of attack are lacking. Here, we show that the level of camouflage achieved through colour adjustments towards the appearance of seaweed habitats is adaptive in reducing predation pressure in the prawn *Hippolyte obliquimanus*. Digital image analysis and visual modelling of a fish predator (seahorse) predicted that brown prawns would be imperfectly concealed against both brown and red seaweed respectively, whereas pink prawns should be well camouflaged only in red weed. Predation trials with captive seahorses (*Hippocampus reidi*), coupled with high-speed video analyses, closely matched model predictions: predation rates were similar for brown prawns between seaweed types, but pink individuals were attacked significantly less on red than brown weed. Our work provides some of the clearest direct evidence to date that colour polymorphism and colour change provides a clear adaptive advantage for camouflage, and also highlights how this can be asymmetric across morphs and habitats (i.e. dependent on the specific background-morph combination).

## Introduction

The study of animal coloration has fascinated evolutionary biologists for centuries and provided important evidence of adaptation and natural selection^1,2^. Colour attributes may modulate individual fitness in many different ways, playing a crucial role in behavioural processes ranging from courtship and mate selection to predator deterrence through visual warning cues^3^. Furthermore, many animals spanning a wide array of taxonomic groups take advantage of their colour patterns for concealment against the surrounding environment^3,4^, mainly by adopting a camouflage strategy known as background matching^4^. In this type of camouflage, better concealed individuals are less frequently detected by visual predators and therefore their survival chances are higher^5^. This is a fundamental prediction of camouflage theory but, despite several emblematic cases consensually considered key examples of natural selection^6–9^, appropriate experimental evidence of the adaptive function of camouflage remains remarkably rare.

A substantial body of previous work has used artificial dummy prey^10,11^ or computer-generated stimuli^12,13^ to test the survival advantage of camouflage in the laboratory or in the field. Other studies, such as the classic example of camouflage and industrial melanism in the peppered moth (*Biston betularia*)^6^, have used correlational evidence, often based on morph-specific recapture rates, or artifical prey targets^9^ to support the hypothesis that better camouflaged individuals are less frequently attacked by predators. More recently, with a better understanding of the anatomy of predator eyes, spectral sensitivity and visual modelling, different studies have estimated how individuals are camouflaged based to the view of predators through the use of spectrometry^14–18^ or digital imagery^19,20^. However, while all these studies comprise important evidence that individuals are efficiently concealed against the substrate, no study has directly quantified how closely differently coloured individuals match their background to predator eyes, and then how matching effectively reduces predation rates in natural conditions. As such, the most basic, yet fundamental prediction of camouflage theory, is still poorly validated^21^.

Camouflage through colour change is commonplace in the animal kingdom and may be achieved over different time scales; from responses of less than a minute, when individuals are moving through a patchy background, to ontogenetic shifts over months or years, accompanying the transition between nursery and adult habitats^21^. In general terms, colour change is basically mediated by different endocrine and cellular processes, usually guided by vision, promoting modifications on the state and abundance of pigment-containing chromatophore cells^21,22^. Physiological colour changes refer to the dispersal or aggregation of pigments within chromatophores and determines patterns of fast colour change, within seconds or minutes, such as those observed in cephalopods^23^ or chameleons^24^. Slower morphological changes over days, weeks or months^21,22^ are more common and imply alterations in the quantity and proportion of chromatophore types and pigment content. Colour-changing species make ideal systems to investigate the adaptive value of camouflage^21^, because they allow testing the importance of colour adjustments of immigrant individuals upon contact with novel habitat, and also whether survival advantages of adjusted individuals are symmetrical between habitats. Despite its potential to unravel important ecological and evolutionary processes, suitable tests of the survival advantage of camouflage in colour-changing species are still rare. Some studies have used vision models to assess changes in concealment^20,25^, but they have not confirmed modelling outcomes with predation trials. Other studies include tethering or predator-exclusion experiments, but they have not modelled prey camouflage to the vision of predators^26,27^.

**Figure 1:**
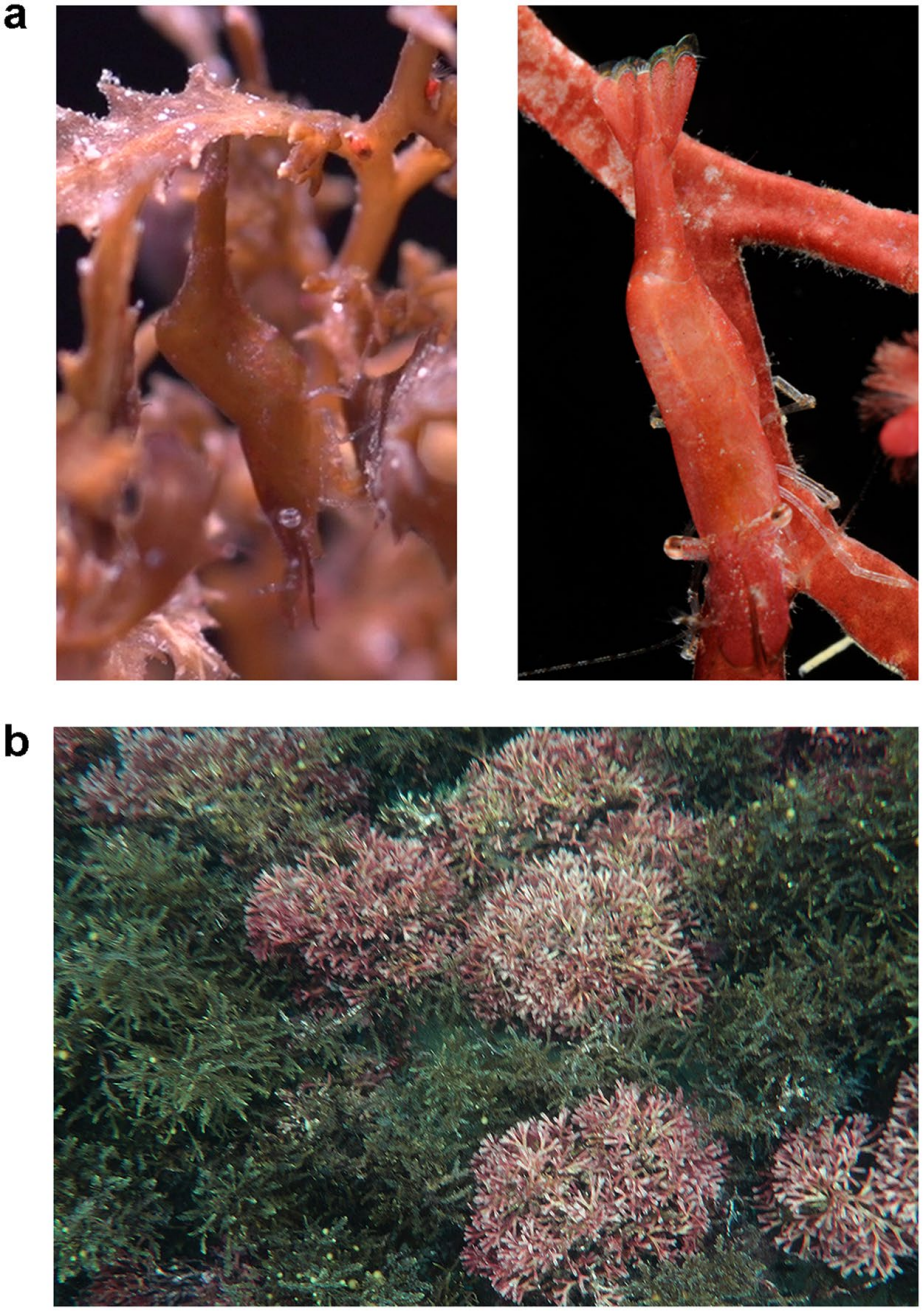
*Hippolyte obliquimanus* colour morphs and seaweed habitats. (**a**) Brown (left) and pink (right) prawns resembling the colour of the brown seaweed *Sargassum furcatum* and the red-pink seaweed *Galaxaura marginata*, respectively. (**b**) *Sargassum* and *Galaxaura* canopies as commonly observed in shallow rocky reefs along the South-eastern Brazilian coast.

The marine prawn *Hippolyte obliquimanus* (Decapoda: Caridae) is a common seaweed-dwelling species found in shallow vegetated areas along the western Atlantic coast, from the Caribbean to Southern Brazil^28^. This species is polymorphic in colour, with individuals presenting homogeneous coloration that can be brown, yellow, green, red or pink, or comprising partially or fully transparent forms marked with stripes or spots^29^. Prawn polymorphism has been thought to function as protective coloration and to provide camouflage against the seaweed types where prawns live. Optimal concealment should be important in reducing both the detection and consumption of prawns by visual fish predators, especially those living in close association with seaweed, such as seahorses, gobies and blennies^30,31^. In Southeast Brazil, prawns exhibiting solid colour patterns on a range of brown to pink tones are commonly found associated with the brown seaweed *Sargassum furcatum* and the red weed *Galaxaura marginata*^29^ (Fig. 1). Both morphs are capable of changing their colour in the direction of their host substrates over a period of a few days^32^, but changes are more remarkable and prawns obtained better concealment when kept in the less intricate red seaweed *Galaxaura*^32^. Although based on colour reflectance alone, holding no relationship with any specific visual system^33^, those results are consistent with the hypothesis that camouflage through colour change is more important in the less structured habitat where shelter is limited (*Galaxaura*), compared to the physically more complex habitat (*Sargassum*) where refuges are more abundant and background matching probably less critical^32^. Although this species is widely distributed along the Central and South America^28^, there are no studies testing whether prawns from other regions and living on substrates of different coloration are also capable of changes to their colour and camouflage against variable backgrounds.

Here we tested the adaptive value of colour change and camouflage in *H. obliquimanus* prawns. First, we described colour variation within and between morphs to test whether ‘pink’ and ‘brown’ individuals can actually be viewed as distinct categories, and compare the colour of prawns and their host seaweed habitats to verify how closely they resemble their background. Based on their likelihood to remain unnoticed by a seahorse predator, which exhibits colour vision and detects prey primarily using visual cues^31,34^, we next quantified the camouflage of prawn morphs on both the host and the alternative seaweed habitat using image analyses and visual modelling. This translates in nature to the capacity of individuals to conceal in primary habitat, where they have remained long enough for colour adjustments to take place, and in secondary habitat shortly upon arrival. Finally, we tested model predictions in a manipulative experiment using real prey and predators. Considering previous results on habitat-specific prawn camouflage based on general colour reflectance^32^, we tested the hypothesis that the survival advantage of camouflage through colour change is dependent on the seaweed habitat, with much reduced detection and predation rates on individuals adjusting their coloration to the red seaweed *Galaxaura* compared to those shifting towards the brown seaweed *Sargassum*.

## Results and Discussion

**Figure 2:**
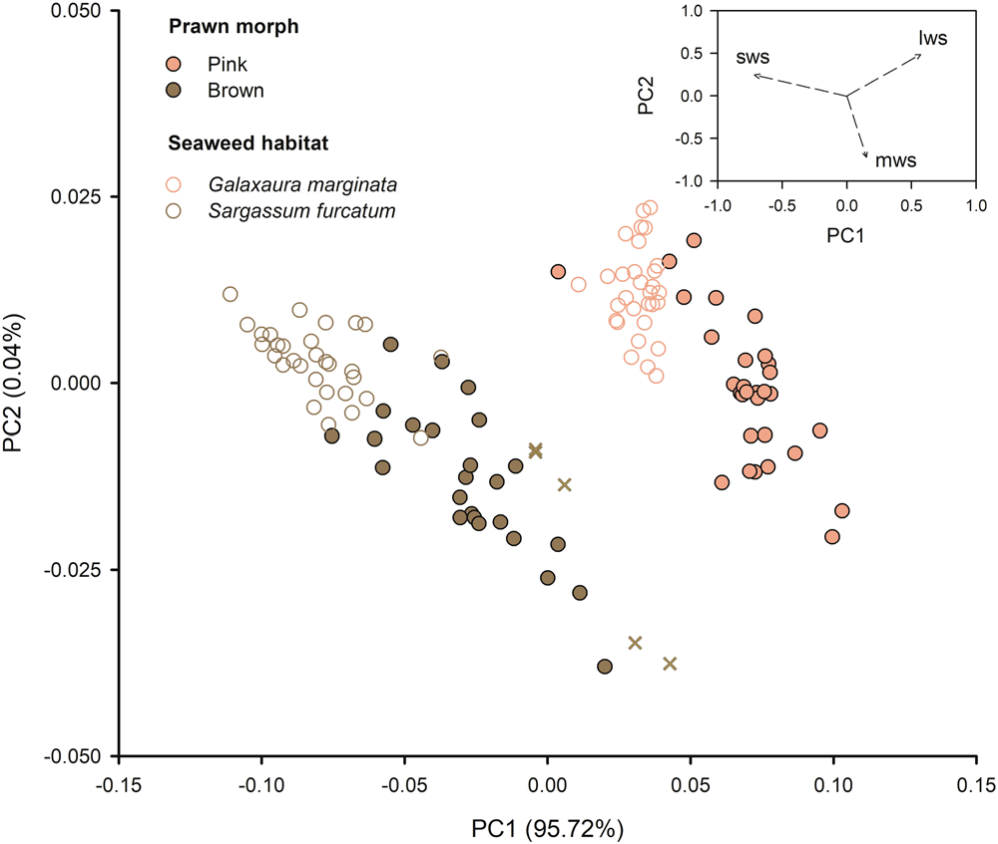
Background resemblance of prawn morphs against seaweeds. Principal Component Analysis applied to seahorse *Hippocampus subelongatus* cone catches showing colour variation of *Hippolyte obliquimanus* colour morphs (‘pink’ and ‘brown’ to the human vision) and seaweed habitats (red *Galaxaura marginata* and brown *Sargassum furcatum*). Percentage values correspond to the total variation explained by each component. The upper-right indent panel indicates that the shortwave colour channel (sws) is the main responsible for the segregation of groups. Brown crosses indicate the few (*n* = 5) cases in which prawn colour resemblance was closer to the alternative rather than to the host habitat colour (all ‘brown’ individuals which were actually closer to *G. marginata*). Sws, mws and lws stand for short, medium and long-wave sensitivity channels.

Our results validate the distinction of brown and pink prawns and their segregation between habitats, reinforcing the need to examine the adaptive value of camouflage separately in brown and red seaweed canopies. Principal component analyses applied on standardised seahorse cone catch values of prawns and seaweed indicate that ‘pink’ and ‘brown’ morphs of the prawn *Hippolyte obliquimanus* are clearly discrete colour entities to both the vision of humans and seahorses, and confirm that prawns tend to adjust their colour to the host seaweed since prawns categorized as pink and brown cohesively clustered with the seaweeds *Galaxaura* and *Sargassum* respectively (Fig. 2). Discriminant function analyses further validated the prawn classification, as all individuals were correctly reassigned to their morphs, and further supported the correspondence of prawn morphs to seaweed species, as 55 out of 60 prawns (92%) were correctly linked to their host weed. The few exceptions were invariably ‘brown’ prawns lying closer to the red *Galaxaura* than to the brown *Sargassum* pattern (crosses in Fig. 2). In fact, the wider spread of brown individuals in Fig. 2 indicates an overall less precise physiological response of prawns acclimating to *Sargassum*, and provides first evidence for less effective camouflage in these individuals compared to prawns established in *Galaxaura*.

**Figure 3:**
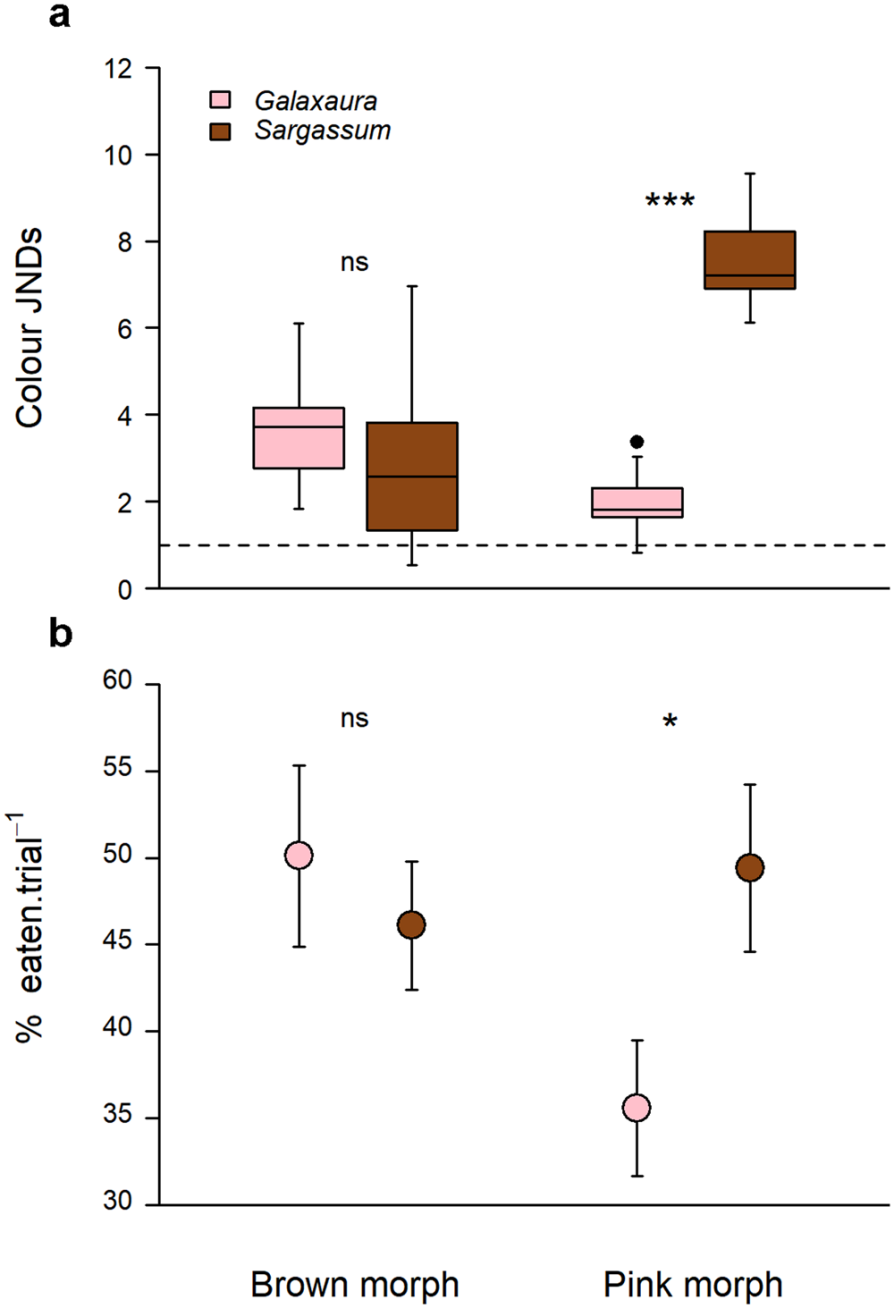
The adaptive value of camouflage in *Hippolyte obliquimanus* prawns. (**a**) Seahorse vision discrimination (as ‘just noticeable differences’; JNDs) of prawn morphs against seaweed habitats. Boxes display medians and inter-quartile ranges (IQRs), whiskers represent lowest and highest values within 1.5∗IQRs, and black filled circles represent outliers. The dashed line (JND = 1) indicates the threshold for visual discrimination of prawns by seahorses. ns: not significant; ***p < 0.001. (**b**) Seahorse predation rates, as the percentage of individuals consumed in 2 h trials (mean ± SE), on brown and pink prawn morphs when placed in *Galaxaura* and *Sargassum* habitats. ns: not significant; *p < 0.05.

Predator discrimination of prawn morphs further suggests that any advantages of camouflage through colour change may be modest in *Sargassum*, but important in *Galaxaura*. Here, we used the discrimination model of Vorobyev and Osorio^35^ for colour and luminance and infer prey detectability based on “Just-Noticeable Differences” (JNDs) to seahorse vision. Briefly, prey are predicted to be discriminated from the background for JND values higher than 1, with detection chances increasing beyond that threshold level, even under unfavourable viewing conditions^36^. Contrasts of colour JNDs between prawns and background habitats are morph-specific, as indicated by the significant interaction term in Table 1. Namely, the colour discrimination of pink prawns in *Galaxaura* (mean JND ± SE; 1.99 ± 0.17) is much lower than in *Sargassum* (7.57 ± 0.28; Fig. 3a), while brown prawns were similarly discriminated in both algal habitats, above the colour detection threshold (3.24 ± 0.40; Fig. 3a). In other words, colour change should lead to superior camouflage and lower detection rates in *Galaxaura* but not in *Sargassum* (see how both prawn morphs and seaweed look like in the view of seahorses in the Supplementary Fig. S1). It is important to note that JND variation was lowest for pink prawns in *Galaxaura* and highest for brown morphs in *Sargassum*, further indicating that improved concealment to seaweed background relies on a precise physiological response leading to a standardised colour pattern. The markedly right-skewed distribution of JND values for brown prawns in *Sargassum* suggests that the poorer camouflage in this habitat is due to the relatively few individuals attaining exceedingly high JNDs (Fig. 3a). Results on luminance contrasts were less informative because they were consistently much higher than detection thresholds across level combinations of factors ‘prawn morph’ and ‘seaweed habitat’ (mean JND ± SE; 6.63 ± 0.62), and therefore are not likely to modulate any predator effects. The significant *p*-level of the interaction term (*p* = 0.046, Table 1) is attributed to morph-dependent habitat differences, with brown prawns showing lower JNDs in *Sargassum* (6.08 ± 1.20) than in *Galaxaura* (9.65 ± 1.41), and pink prawns showing similar JNDs between seaweed habitats (5.41 ± 0.74). Although being consistently lower for pink prawns on both habitats, all luminance contrasts were much higher than the putative threshold for detection, indicating that seahorses probably did not use this channel for detecting their prey and primarily base their hunting behaviour on colour cues^34^. However, we note that the achromatic version of the receptor noise model is much less tested than the chromatic model (the original model originally disregarded achromatic information altogether)^35^, and the mechanism of achromatic perception in fish is often poorly known and variable. Therefore, caution should be used with interpreting the overall magnitude of the luminance JND values. Additional behavioural experiments are necessary to understand the importance of both chromatic and achromatic signals in the visual repertoire of this predator^37^.

**Table 1:** Summary results of prawn camouflage against seaweed backgrounds based on seahorse vision.

Source of variation	*df*	Colour JNDs			Luminance JNDs		
		*MS*	*F*	*p*	*MS*	*F*	*p*
Prawn morph – M	1	35.7	21.50	<0.001	92.0	4.42	0.040
Seaweed habitat – S	1	86.6	52.08	<0.001	20.4	0.98	0.327
M × S	1	150.4	90.43	<0.001	86.4	4.15	0.046
Error	56	1.7			20.8		
		Cochran’s *C* = 0.541; *p* < 0.01			Cochran’s *C* = 0.356; *p* > 0.05		

Results of two-way analyses of variance testing differences in “just-noticeable differences” (JNDs) for colour and luminance measurements, according to combinations of *Hippolyte obliquimanus* colour morphs (‘brown’, ‘pink’) and seaweed backgrounds (*Galaxaura marginata*, *Sargassum furcatum*). Cochran’s *C*: Cochran statistic testing variance heterogeneity.

**Figure 4:**
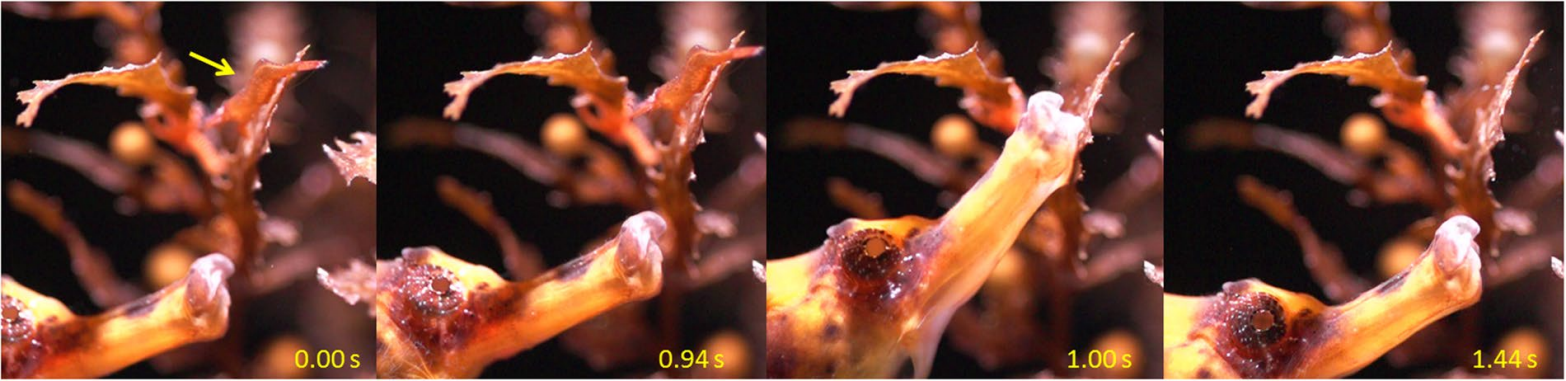
Sequence of still images from high-speed video footage (480 fps), over less than 1.5 s, showing a seahorse preying on a prawn camouflaged on brown seaweed *Sargassum furcatum*. The yellow arrow indicates the prawn position in the first frame. Note that the attack took shorter than 0.06 s (frame 2 to 3).

Results of predation trials closely corresponded to colour JND modelling, thus supporting the adaptive value of camouflage through colour change as a mechanism to reduce predation rates on the prawn *Hippolyte obliquimanus* (Fig. 3). Habitat-dependent predation on prawn morphs is backed by the significance of the interaction term of the linear model examined (Table 2): seahorses *Hippocampus reidi* equally preyed on brown prawns held at the two seaweed habitats (mean ± SE; *Sargassum*: 46.1 ± 3.7%; *Galaxaura*: 50.1 ± 5.2%), but predation rates on pink individuals were reduced to almost 35% in *Galaxaura* compared to *Sargassum* (*Sargassum*: 49.4 ± 4.8%; *Galaxaura*: 35.6 ± 3.9%), indicating that colour change towards the background was efficient in the red but not in the brown seaweed environment (Fig. 3b). It is important to note that in spite of their much higher JNDs (Fig. 3a) pink prawns on *Sargassum* were eaten at similar rates than brown prawns on either habitat (Fig. 3b), suggesting that detection and predation rates would be high and fairly constant at JNDs over 3 or 4 (i.e. beyond the detection threshold). Interestingly, consumption rates were very consistent among seahorse individuals, as indicated by the non-significant random factor ‘seahorse ID’ nested in the morph*habitat interaction (Table 2). Positive effects of colour adjustments on prey survival may thus be pervasive, dampening any potential behavioural syndromes underlying individual-based differences among predators^38,39^. Consistent results among individual predators probably reflect specialized hunting techniques, involving a very specific pattern of prey spotting, approaching and striking common to all seahorse individuals (Fig. 4). High-speed video recordings (480 fps) taken during experimental trials confirmed that seahorses use primarily visual cues for prey detection, taking on average 4.28 ± 0.82 s (mean ± SD) to strike after first visual contact (Supplementary Video S1). Once detected, seahorses move slowly without losing eye contact until they reach a distance to prey that can be covered during a very fast strike (less than 0.063 s; Fig. 4). Still, our observations show that strikes involve an upward rotation of the head (frame 2 to 3), slightly increasing the path travelled by the mouth as revealed by models of seahorse feeding biomechanics^40^. According to these authors, an extended strike distance allows seahorses to probe a larger volume of water and hence locate prey more precisely, which could explain the very high percentage of successful attacks (90%) observed in our trials.

**Table 2:** Summary results of seahorse predation on prawn colour morphs.

Source of variation	Predation rate			
	*df*	*MS*	*F*	*p*
Prawn morph – M	1	0.031	3.39	0.103
Seaweed habitat – S	1	0.020	2.22	0.174
Seahorse ID (M × S)	8	0.009	0.43	0.888
M × S	1	0.076	8.38	0.020
Error	24	0.021		
	Cochran’s *C* = 0.324; *p* > 0.05			

Results of mixed-model analysis of variance testing contrasts of seahorse *Hippocampus reidi* predation rates on prawn *Hippolyte obliquimanus* colour morphs maintained in different seaweed habitats (as percentage of individuals consumed by seahorses over 2 h trials). The factors ‘prawn morph’ and ‘seaweed habitat’ are fixed, while ‘seahorse ID’ is random and nested in the interaction of main factors. Cochran’s *C*: Cochran statistic testing variance heterogeneity.

In this study we present novel evidence showing the adaptive value of camouflage through colour change. A wide range of recent studies have tested how types and levels of camouflage affect detection, but predominantly using artificial (human-made) ‘prey’ presented to human and other animal observers^10,41^. Furthermore, while iconic studies of the peppered moth quantified morph-specific survival advantage in different habitats^6^, and recent studies of wild birds have shown that camouflage level correlates with survival in the field^19^, no study has yet directly demonstrated that camouflage level, to predator vision, directly influences individuals’ survival chances. Here, the visual model we used closely predicted specific camouflage success for each *H. obliquimanus* colour morph on each seaweed background in terms of colour discrimination/detection to a seahorse predator. Therefore, our study is the first to quantitatively demonstrate that predation risk in an animal is directly related to predator-perceived levels of camouflage, and concurrently validates widely used but seldom tested models of visual discrimination. Although focusing in a specific seahorse predator, which exhibits colour vision^34^ and uses visual cues to detect prey^31^ (Fig. 4, Supplementary Video S1), our results should be generalizable to other fish potentially hunting *H. obliquimanus*, including gobies, blennies and pinfish species which are frequently found associated with *Sargassum* and *Galaxaura* canopies^30,42^. There is no specific information regarding the visual system or the existence of colour vision in these alternative predators, but studies on similar species have suggested that most of them use colour cues to detect prey^37,43–46^ and therefore would likely exhibit similar behaviour to seahorses and be potentially deceived by prawn camouflage.

In our study we found that the survival advantage of camouflage through colour change is asymmetric across different habitats. Colour concealment was shown to be adaptive for prawns shifting to pink in *Galaxaura* but not for prawns changing to shades of brown in *Sargassum*; a result consistent to our initial predictions. Adequate shelter and extensive foraging grounds provided by the more intricate architecture of *Sargassum* and accompanying epiphytic algae^47,48^ may be more important than concealing coloration to maintain high prawn densities in the brown weed habitat^29^. Differently, lower prey density and reduced shelter supply - two conditions known to increase per-capita predation pressure^49,50^ - make lower prey detection rates critical in the less complex *Galaxaura* canopy. In summertime, *Sargassum* blooms, becomes primary habitat and hosts very large prawn aggregations^29^, but by winter time the brown-weed have decayed^51,52^ and the perennial *Galaxaura* becomes a more important habitat. Fast colour change allowing camouflage in the red weed canopy^32^ may be therefore of paramount importance by increasing survival rates of overwintering individuals and hence ensuring population stability through time.

In conclusion, by integrating the more recent area of image analysis and visual modelling with classical behavioural experiments our study highlights an important future avenue of research in both sensory and behavioural ecology. The results we obtained represent a fundamental starting point for understanding the adaptive value of camouflage – one of the most common anti-predator strategies observed in nature – for many different species. In addition, colour change for camouflage is widespread in nature, being common in animals from both terrestrial and aquatic habitats^21^, which permits the generalization of our findings to different species living on heterogeneous habitats, such as many insects^53^, crabs^54–56^, fish^25,57^ and lizards^58^. It is important to appreciate, however, that both colour change and camouflage may differentially affect the survival of individuals in each of the different habitats where they live, since each background type will exhibit specific requirements that may change the close relationship between animal and substrate coloration.

## Methods

### Field sampling

Seaweeds *Sargassum furcatum* and *Galaxaura marginata* were collected by skin diving in the vicinities of the Centre for Marine Biology, São Sebastião, SP, Brazil (23°49′40′S; 45°25′22′W) during the spring of 2015 and summer of 2016. Prawns were sorted from seaweeds (as in^29^) and visually classified as brown or pink morphs, which proved to be a simple method for an accurate assignment^32^ (Fig. 2). Before being used in experiments, prawns and seaweeds were kept in indoor tanks (30 × 20 × 10 cm) at ambient temperature (~27 °C), supplied filtered running seawater and aeration. A random set of prawn and seaweed samples was separated for image analyses and visual modelling to measure the potential of prawn camouflage against algal habitats. Another set was used for predation experiments to test predictions of modelling results.

### Prawn camouflage

Pieces of seaweed and living prawns (*n* = 30 for each seaweed species and prawn morph) were photographed in an acrylic chamber (5 × 5 cm) using a Nikon D80 digital camera, coupled with a Nikkor 60 mm lens and a UV-blocker filter (62 mm, Tiffen, USA). The camera was set up to capture only visible light (400 to 700 nm) because objects exhibited low overall UV reflection (as observed in images acquired with a UV-sensitive camera), and because fish preying on prawns are likely less sensitive to UV light^43,59^. Images were taken in RAW format, with manual white balancing and fixed aperture settings to avoid over-exposition^60^, and included black (7.5%) and white (91%) Spectralon reflectance targets (Labsphere, Congleton, UK) following the current standard procedure^61^. Illumination was provided by one human visible Colour Arc Lamp (70 W, 6500 K Iwasaki), coupled to a polytetrafluoroethylene diffuser cylinder around the photography chamber to ensure even lightning. Images were successfully linearized (R² ≥ 0.997 for all camera channels), using the curves modelled from eight Spectralon reflectance standards (from 2 to 99% reflectance) to correct for camera non-linear pixel responses to light intensity^60,61^. Photographs were equalized for changes in light conditions using 7.5% and 91% standards and saved as 32-bit multispectral images. All routines were performed using customized functions in the ImageJ software^61,62^.

Prawn and seaweed colour was analysed based on a seahorse vision model, since seahorses are abundant in seaweed beds^63^ and commonly prey on caridean prawns^64^, including *H. obliquimanus*^31^. Since there is no information on the visual system of the local seahorse predator *Hippocampus reidi*, the spectral sensitivities of the closely related species *Hippocampus subelongatus*^59^ were used for modelling. We assumed the visual capacity of the two seahorse species are similar as they both live in similar green-water vegetated habitats^59,65^. *H. subelongatus* has spectral peaks for single cones at 467 nm (shortwave sensitivity, SWS), and for double cones at 522 nm (mediumwave, MWS), 537 nm (medium-longwave, M-LWS), and 560 nm (longwave, LWS)^59^. A 50% light transmission cut-off at 425 nm was incorporated^59^, and a D65 standard irradiance spectrum was used as a measure of incident illumination^66^, compatible to the restricted shallow-water environment, of only a few cm, where predator-prey visual interactions take place (Fig. 4). We assumed that colour vision is encoded by independent spectral channels in double cones (DCs), as reported for the reef fish *Rhinecanthus aculeatus*^45^. Compared to other fish which have only one or two pigments in their DCs^45,67^, the seahorse *H. subelongatus* exhibits an unusual DC configuration, with three different cone types accounting for the medium-long wave sensitivity^59^. We thus assumed that *H. subelongatus* has a trichromatic visual system, but still modelled colour vision as encoded by SWS single cones plus LWS DCs, and either MWS DCs (‘Model 1’) or M-LWS DCs (‘Model 2’). We only report results for ‘Model 1’ because outputs of both models were very similar (Supplementary Table S1). Tetrachromatic vision was discarded because similar MWS and M-LWS cone types were present in DCs, strongly suggesting that one of them is used for luminance (i.e. achromatic) contrast^59^. Polynomial mapping was used to convert images from the camera colour space^60,68^ into values of seahorse cone catches, closely corresponding to spectrometry techniques^19,20,25,61^. Before building the model, we calculated the spectral sensitivity curves of our equipment^20,69^, and obtained the following sensitivity range and spectral peaks: SW; 400–550 nm (peak 472 nm), MW; 420–620 nm (peak 534 nm), LW; 550–700 nm (peak 596 nm).

Visual modelling resulted in multispectral images that were used to estimate photon catch values for each colour channel in the selected regions of interest (ROIs; prawn carapace and abdomen, from the region behind the eyes to the end of the third abdominal somite, avoiding the stomach region, and seaweed fronds). A principal component analysis (PCA) on the covariance matrix of the standardized cone data was used to visualise colour differences between morphs and backgrounds and to determine the channels responsible for clustering. Prawn and seaweed PC scores (PC1 and PC2) were used to create discriminate functions to, respectively, confirm morph classifications and validate the correspondence of morphs to seaweed species. The ‘lda’ function from the MASS library in R^70^ was used to run discriminant function analyses. A widely implemented log-linear form of colour discrimination model^35^, which assumes limitation by receptor noise, was used to predict chromatic and achromatic perception as “just noticeable differences” (JNDs). Since behavioural data backing visual discrimination is lacking for *H. subelongatus*, we used a conservative and frequently adopted Weber fraction value (0.05) for the most abundant cone type^66^, and set cone proportions to LWS = 0.44, MWS = 1.00, M-LWS = 0.89 and SWS = 0.56^59^. Colour detection by predators is expected at JNDs higher than 1.00^36^. We then calculated colour and luminance contrasts in single prawn-seaweed random pairings, resulting in 15 independent JNDs for each prawn morph/seaweed species combination. Colour and luminance JNDs were analysed separately using a 2-way ANOVA, in which factors ‘prawn morph’ (brown or pink) and ‘seaweed type’ (*Sargassum* or *Galaxaura*) were fixed and orthogonal. Variances remained heterogeneous for colour JNDs even after log transformation. Still, we proceed with the analysis using raw data because this was a balanced design with a large sample size (*n* = 15), which makes the test robust to variance heterogeneity^71^. The Student-Newman-Keuls (SNK) procedure was used for *a posteriori* comparisons.

### Laboratory predation trials

There were different reasons to select seahorses as ideal model predators in this study. First, seahorses are specialised consumers of seaweed-dwelling invertebrates, curling their tail around weed thalli or holdfasts and ambushing prey upon visual detection^63^. Second, caridean prawns have been ranked first or second in seahorse diet^72,73^. Regarding our focal species, the prawn *Hippolyte obliquimanus* is heavily consumed by *Hippocampus reidi*, preferring this prey to amphipods and brine shrimp^31^. Third, *H. obliquimanus* and *H. reidi* are common species in our study region^29,74^ and therefore the predator-prey interaction addressed here should be quite frequent at the sampling area.

A set of ten cubic aquaria (25 × 25 × 25 cm), supplied a thin layer of natural sand covering the bottom and constant flow of 5-µm filtered seawater, was maintained in natural temperature (26.5 °C ± 1.1) and salinity (31.1 ± 0.7) conditions. Five of these aquaria were used to maintain stocks of freshly collected seaweeds, prawns and seahorses, and the other five were used for experimental trials. Prawn stocks were kept with their original plant hosts (‘brown’ prawns on *Sargassum* and ‘pink’ prawns on *Galaxaura*). Three non-reproductive *H. reidi* individuals (S1: female, height 11.4 cm; S2: female, height 10.6 cm; S3: male, height 11.4 cm) were collected by snorkelling from seaweed meadows in the same area (ICMBio-approved license #55633-1) and kept in individual tanks where they were fed *ad libitum* a variety of seaweed-dwelling invertebrates. Predation trials were carried out under natural daylight in aquaria provided with a clump of either *Sargassum* or *Galaxaura* (approx. 50 ml), devoid of any mobile epifauna after brief immersion in freshwater. In each tank, 20 individuals of either the brown or pink morph were included and left to acclimatize for 10 minutes before the addition of a single seahorse, initially caged in a 5 mm mesh-bag. After 20 minutes, when all prawns had settled on seaweed, the predator was released and left in tanks for 2 hours. Predation rate was calculated as the proportion of prawns that were consumed until the end of the trial. A maximum of two experimental aquaria were run at the same time and combinations among levels of factors ‘prawn morph’, ‘seaweed habitat’ and ‘seahorse ID’ were randomly replicated in time, three times, summing 36 trials over 1.5 months. The tank used in each trial was also randomly chosen to avoid potential artefacts due to uncontrolled spatial variation of any physical variables within laboratory space. We also certified that seahorses were left without food for at least 20 hours before their use in trials, ensuring that complete gastric evacuation has occurred^31^. In some trials (*n* = 10) we used a high-speed camera (Sony NX-FS700R, coupled with a Nikkor 60 mm lens, capturing images at 480 fps) to record seahorse hunting behaviour. All experimental procedures complied with Brazilian ethical standards.

Predation rate was analysed using a specific ANOVA model in which the factors ‘prawn morph’ (brown and pink) and ‘seaweed habitat’ (*Sargassum* and *Galaxaura*) were considered fixed and orthogonal. ‘Seahorse ID’ (S1, S2 and S3) was included as a random factor, nested in the interaction of main factors, allowing proper replication and a test for the generality of predation effects. As for JND comparisons, we used the SNK post hoc test to further examine significant sources of variation.

### Ethics

Collection of seahorses and their maintenance in the laboratory together with their use in the experiments complied with Brazilian ethical standards and were licensed accordingly [‘Instituto Chico Mendes de Conservação da Biodiversidade’ (ICMBio), license number #55633-1].

## Electronic supplementary material

Video S1

Supplementary information

## Data Availability

The data generated and analysed during the current study are available from the corresponding author on request.
