# Supplementary material for: The adaptive value of camouflage and colour change in a polymorphic prawn
Source: Sci Rep. 2018 Oct 30;8:16028. doi: 10.1038/s41598-018-34470-z (PMC6207773; doi:10.1038/s41598-018-34470-z)
Supplement: Supplementary file 2 — Supplementary information [file 41598_2018_34470_MOESM2_ESM.pdf]

Supplementary information

The adaptive value of camouflage and colour change in a polymorphic prawn

Rafael Campos Duarte[a,b,*], Martin Stevens[c], and Augusto Alberto Valero Flores[a]

a – Centro de Biologia Marinha, Universidade de São Paulo. Rod. Manoel Hypólito do Rego, km 131.5, São Sebastião, SP, Brazil. 11612-109.

b – Programa de Pós-Graduação em Biologia Comparada, Faculdade de Filosofia, Ciências e Letras de Ribeirão Preto, Universidade de São Paulo, Ribeirão Preto, Brazil.

c – Centre for Ecology and Conservation, University of Exeter, Penryn Campus, Penryn. TR10 9FE, UK.

* Corresponding author. E-mail: rafaduarte87@usp.br

Table S1. Summary results of seahorse visual modelling. Results of the two-way analyses of variance testing differences in "just-noticeable differences (JND), derived from two visual modelling scenarios (see Methods), according to combinations of *Hippolyte obliquimanus* colour morphs (brown or pink) and seaweed backgrounds (*Galaxaura marginata* and *Sargassum furcatum*) (upper table). The mean JND values and respective 95% confidence intervals (lower and upper bounds) for the different combinations between factors are shown in the lower table. Note that both models resulted in overlapping JND 95% confidence intervals for all factor combinations.

		**Model 1**			**Model 2**		
Source of variation	*df*	*MS*	*F*	*p*	*MS*	*F*	*p*
Prawn morph (M)	1	35.747	21.50	***	26.144	17.25	***
Seaweed habitat (S)	1	86.599	52.08	***	177.966	117.40	***
M x S	1	150.363	90.43	***	165.274	109.02	***
Residual	56	1.663			1.516		

	**Model 1**			**Model 2**		
		95% CI			95% CI	
Factor combinations	mean	lower bound	upper bound	mean	lower bound	upper bound
Brown *Galaxaura*	3.621	2.963	4.279	3.858	2.998	4.718
Brown *Sargassum*	2.857	1.806	3.908	3.983	3.262	4.704
Pink *Galaxaura*	1.998	1.623	2.373	1.859	1.477	2.241
Pink *Sargassum*	7.567	6.964	8.170	8.623	7.947	9.299

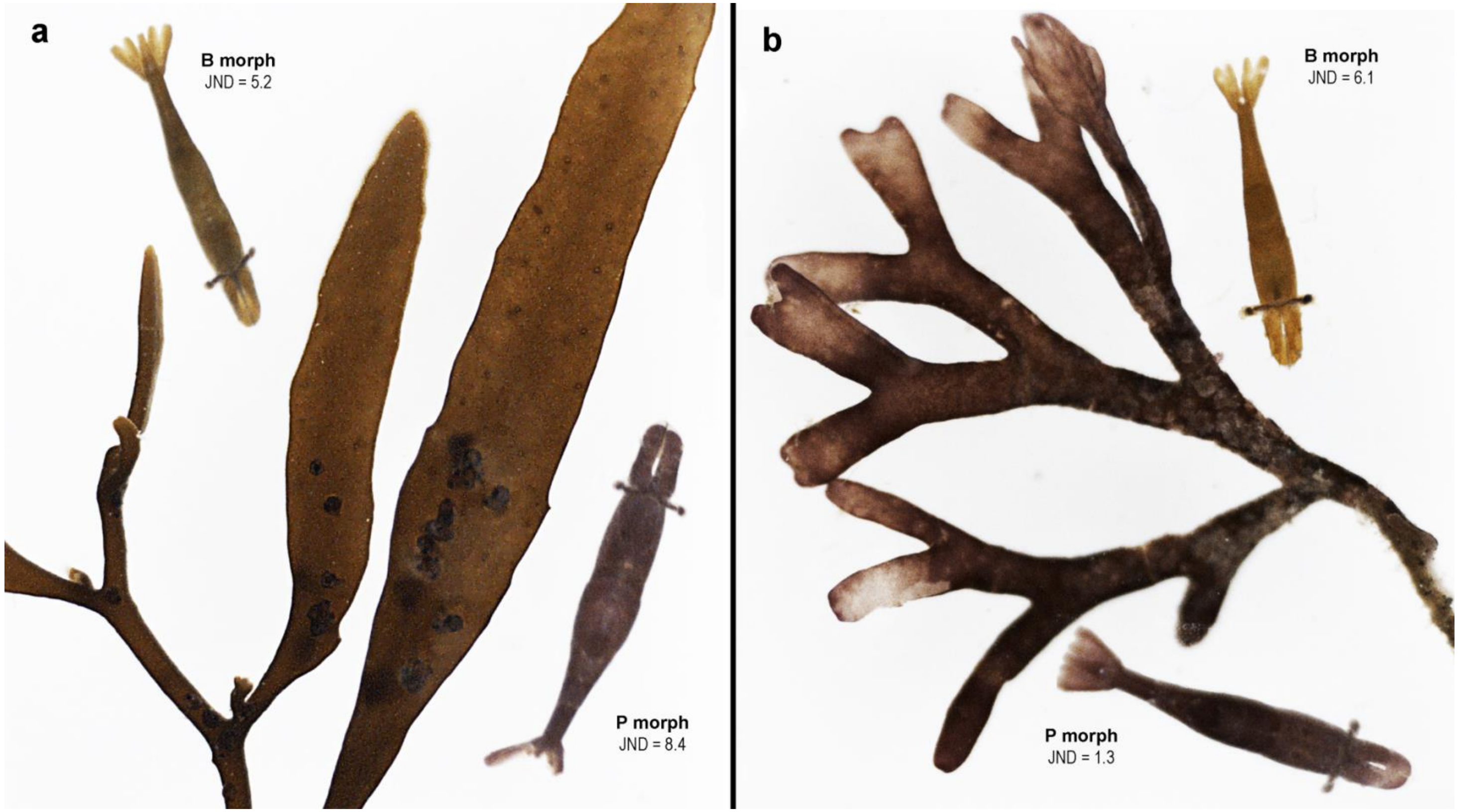

Figure S1 – Differential camouflage of *Hippolyte obliquimanus* colour morphs on the seaweeds *Sargassum furcatum* and *Galaxaura marginata* to the vision of the seahorse *Hippocampus subelongatus*. False-colour seahorse images of brown (B) and pink (P) prawns on *Sargassum* (**a**) and *Galaxaura* (**b**) seaweeds are made from a combination of the blue, green and red channels (corresponding to the mapped shortwave, mediumwave and longwave images of seahorse receptors, respectively). Here, resulting images were square-root-transformed from linear cone-catch photos only for display purposes only. Average JND values resulting from comparing each prawn to the respective seaweed using the Vorobyev-Osorio model are also shown (see main text).
